# Systematic Review of the Association Between Trauma Severity and Postinjury Symptoms of Depression

**DOI:** 10.1007/s00268-022-06750-3

**Published:** 2022-09-29

**Authors:** Yvonne Versluijs, Thomas W. van Ravens, Pieta Krijnen, David Ring, Inger B. Schipper

**Affiliations:** 1 Department of Trauma Surgery Leiden, University Medical Center, P.O. Box 9600, 2300 RC Leiden, the Netherlands; 2 Department of Surgery and Perioperative Care Dell Medical School, The University of Texas at Austin, 1701 Trinity Street, Austin, TX 78705 USA

## Abstract

**Background:**

Greater symptoms of depression are associated with greater symptom intensity during recovery from musculoskeletal injury. It is not clear that more severe trauma is associated with greater symptoms of depression as one might expect. The goal of this study was to systematically review the existing evidence regarding the association of Injury Severity Score (ISS) with symptoms of depression during recovery from musculoskeletal injury.

**Methods:**

Two independent reviewers used PubMed and Embase to identify studies that measured both ISS and symptoms of depression. Among the 17 studies satisfying inclusion criteria, 5 studies assessed the correlation of symptoms of depression and ISS on their continuum; 3 studies compared the mean of symptoms of depression for people above and below a specific ISS level; five compared mean ISS above and below a threshold level of symptoms of depression; and four compared dichotomized ISS and dichotomized depression. Four of the 17 evaluated factors associated with symptoms of depression in multivariable analysis.

**Results:**

In bivariate analysis, 12 of 17 studies (71%) found no association between ISS level and symptoms of depression. Three studies found a bivariate association that did not persist in multivariable analysis. Two studies reported slight associations in bivariate analysis, but did not perform multivariable analysis.

**Conclusions:**

The knowledge that symptoms of depression are common during recovery, in combination with the finding of this review that they have little or no relationship with injury severity, directs clinicians to anticipate and address mental health during recovery from physical trauma of any severity.

## Introduction

Recovery from traumatic injury is traditionally assessed in terms of physical measures such as range of motion and survival [1]. The emotional and social (role security and livelihood) damage merit as much attention as the structural damage [1–4]. Feelings of despair (symptoms of depression) are common after traumatic injury and are characterized by a persistent state of low mood that affects and is affected by patients’ thoughts, behavior, feelings, and social security [1–3, 5–7]. In a meta-analysis, the incidence of a threshold level of symptoms of depression after musculoskeletal injury was found to be 33%, compared to about 7% of the general adult population in the United States [8].

Greater symptoms of depression are associated with greater symptom intensity and greater magnitude of incapability after musculoskeletal injury and surgery [3, 7, 9–11]. Greater symptoms of depression are also associated with adverse events, more limited participation in rehabilitation activities, and delayed return to important life roles such as work [3, 7, 9, 10, 12]. In countries that make liberal used of opioids, continued request for opioids after the body’s initial healing is established is associated with greater symptoms of depression symptoms, regardless of injury severity [13]. The exact psychopathologic mechanisms of developing symptoms of depression is unknown. There is a large individual difference in the way a trauma is processed and its meaning for the person's view of himself, the world, and the future [14]. Initial symptoms such as sense of hopelessness, emotional numbing and sleep disturbance may lead to depression by hampering the person’s ability to generate adaptive beliefs and solutions to problems [14].

In spite of these notable and consistent relationships, clinician awareness and action on mental health opportunities is limited and relatively few patients receive the psychological support they need [1, 7, 12]. Also, when clinicians are referring patients to health services, they face challenges such as long wait times [15]. Once manifested, major symptoms of depression are challenging to treat [3]. One might intuitively expect that higher trauma severity is associated with greater symptoms of depression, what may lead to even less recognition of depressive symptoms among minor trauma patients. The Injury Severity Score (ISS) is a commonly used scoring system for assessing trauma severity in patients with multiple injuries. If trauma severity, measured with ISS, is associated with greater symptoms of depression, trauma severity scores could be used more easily to trigger involvement of mental health professionals. If not, then surgeons and their teams should develop routine and inclusive methods for diagnosing and treating mental health opportunities among all trauma patients.

This systematic review summarized the existing evidence regarding association of symptoms of depression with the Injury Severity Score (ISS) during recovery from non-neurological traumatic injury.

## Materials and methods

This systematic review was performed according to the ‘Preferred Reporting Items for Systematic Reviews and Meta-Analysis’ (PRISMA) guideline [16]. Review protocol exists and is retrievable. Study selection and data extraction were independently performed by two reviewers. Disagreement between the reviewers was resolved by discussion.

### Study selection

A search was performed in Pubmed and Embase on 19st of February 2021 for relevant studies published since 2010. The search strategy was composed by an experienced medical librarian and included various synonyms for musculoskeletal trauma, depression, and injury severity score (Appendix 1). Results of the database search were downloaded into Covidence, an online tool for primary screening and data extraction in systematic reviews. Duplicate articles were removed. Articles were independently screened on title and abstract.

The full text of the remaining articles was read and eligibility based on the following criteria: (1) traumatic injury, (2) patients aged 18 or older, (3) the association between symptoms of depression on any measure and injury severity score (ISS) was analyzed and reported, and (4) article written in English. We excluded studies involving patients with isolated traumatic brain injury (TBI) because cognitive impairment and neurobehavioral symptoms may affect or preclude assessment of the mental status.

### Data extraction

From the included articles, data were extracted on study characteristics; year of publication, country, study design, number of included patients, follow-up time, patient age and patient sex. Data of interest were the ISS (study population mean and definition for analysis), depressive symptoms (measurement tool, mean score or percentage with a threshold of symptoms of depression, and the definition for analysis), and the results of univariable and multivariable analyses of the relationship between ISS and depression.

The ISS is a commonly used scoring system for assessing trauma severity in patients with multiple injuries [17]. Scores of < 9 are representing mild injuries, 9–15 moderate and ≥ 15 severe [17]. A meta-analysis was not performed due to variation in the definition of depression and the use of different statistical methods across the included studies*.*

### Risk of bias and quality assessment

The risk of bias in the included studies was scored using the methodological items for non-randomized studies (MINORS) [18]. This tool consists of eight items regarding the design of non-comparative studies. Each item is scored as 0 (not reported), 1 (reported but inadequate) or 2 (reported and adequate). The maximum score is 16, representing no risk of bias.

## Results

### Study selection and patient population

**Fig. 1:**
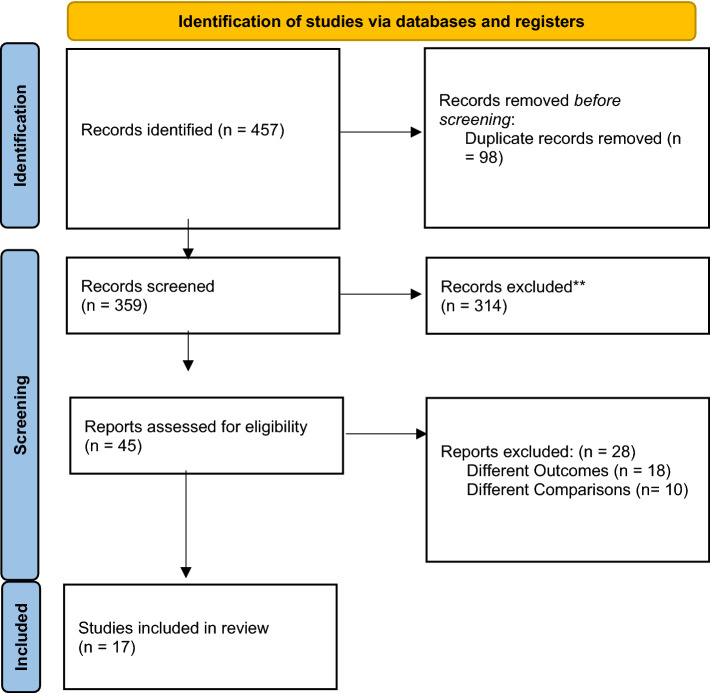
PRISMA flow diagram

The search in Pubmed and Embase resulted in 457 studies (Fig. 1). After exclusion of 98 duplicates, 359 studies remained, of which 314 articles were excluded based on title and abstract. Twenty-eight of the remaining 45 studies were excluded because the association between ISS and symptoms of depression was not addressed, resulting in 17 studies [19–35] suitable for this systematic review. One was a retrospective and 16 were prospective cohort studies (Table 1). Together, the 17 studies included 5,969 patients. The number of per study included patients varied between 32 [36] and 1,166 [20]. The mean or median age varied between 25 and 48 years [21, 31]. The majority of patients were men (weighted mean 70%; range 56% to 100%). Five studies assessed the correlation of symptoms of depression and ISS on their continuum; three studies compared the mean of symptoms of depression for people above and below a specific ISS level; five studies compared mean ISS above and below a threshold level of symptoms of depression; and four compared dichotomized ISS and dichotomized depression. Four of the 17 also evaluated factors associated with symptoms of depression in multivariable analysis.

**Table 1:** Study and patient characteristics

Author	Year of publication	Country	Study design	Numbers of included participants	Follow-up time	Age in years (mean ± SD or median [IQR])	Male patients
Archer et al	2016	United States	Prospective Cohort study	213	12 months	44 ± 16	56%
Baecher et al	2018	Australia	Prospective Cohort study	1166	12 months	38 ± 14	74%
Biçen et al	2021	Turkey	Retrospective Cohort study	81	9 months	30 ± 8	100%
Boals et al	2017	United States	Prospective Cohort study	460	0, 3, 6 and 12 months	44 ± 17	66%
Bocci et al	2016	Italy	Prospective Cohort study	32	12—24 months	38 [27–51]	91%
Gouweloos et al	2016	the Netherlands	Prospective Cohort study	121	2 and 9 months	40 ± 13	70%
Halvachizadeh et al	2020	Switzerland	Prospective Cohort study	337	Mean 29 years	25 ± 12	75%
Jackson et al	2011	United States	Prospective Cohort study	173	12 months	43 ± 17	57%
Kumar et al	2020	India	Prospective Cohort study	190	During hospitalization	Mean 34	79%
Rainey et al	2014	United States	Prospective Cohort study	110	0 and 12 months	46 ± 19	60%
Schweininger et al	2015	Australia	Prospective Cohort study	1017	0, 3 and 12 months	38 ± 14	74%
Shelley et al	2020	United States	Prospective Cohort study	506	12 months	48 ± 17	56%
Shih et al	2010	Canada	Prospective Cohort study	677	6 and 12 months	Mean 34	78%
Skogstad et al	2014	Norway	Prospective Cohort study	181	12 months	Mean 42	59%
vanDelft-Schreurs et al	2014	the Netherlands	Prospective Cohort study	281	15–53 months (mean 3 years)	47 ± 19	69%
Vincent et al	2018	United States	Prospective Cohort study	101	12 weeks	44 ± 16	59%
Wu et al	2017	China	Prospective Cohort study	323	During hospitalization	44 ± 13	66%

### Quality assessment

The MINORS score varied between 8 and 13 (Table 2). The quality assessment revealed a risk of bias for all included studies. For example, all studies had a risk of bias, because loss to follow-up was more than 5% or not reported at all.

**Table 2:** Risk of bias of selected studies, assessed with the Methodological Index for Non-Randomized Studies (MINORS) tool

MINORS scores	1	2	3	4	5	6	7	8	Total
Archer et al	2	0	2	2	0	2	1	0	9
Baecher et al	2	1	2	2	0	2	1	0	10
Biçen et al	2	2	1	2	0	1	1	0	9
Boals et al	2	2	2	2	0	2	1	0	11
Bocci et al	2	2	2	2	0	1	1	0	10
Gouweloos et al	2	2	2	2	0	2	1	0	11
Halvachizadeh et al	2	0	2	2	0	2	1	0	9
Jackson et al	2	2	2	1	0	2	1	0	10
Kumar et al	2	2	2	2	0	1	0	1	10
Rainey et al	2	1	2	2	0	2	1	0	10
Schweininger et al	2	1	2	2	0	2	1	0	10
Shelley et al	2	0	2	2	0	2	1	0	9
Shih et al	2	1	2	2	0	2	1	0	10
Skogstad et al	2	2	2	1	0	2	1	0	10
vanDelft-Schreurs et al	2	2	2	2	0	1	1	0	10
Vincent et al	2	2	2	2	0	2	1	2	13
Wu et al	2	1	2	2	0	1	0	0	8

The items are scores 0 (not reported), 1 (reported but inadequate) or 2 (reported and adequate). Maximum score 16

1. A clearly stated aim

2. Inclusion of consecutive patients

3. Prospective collection of data

4. Endpoints appropriate to the aim of the study

5. Unbiased assessment of the study endpoint

6. Follow-up period appropriate to the aim of the study

7. Loss to follow-up less than 5%

8. Prospective calculation of the study size

### Injury severity score

The ISS varied widely, due to the inclusion criteria (Table 3). Bocci et al. [36], Halvachizadeh et al. [37], Jackson et al. [38] and van Delft et al. [39] only included patients with severe trauma (ISS > 15). All other studies included both patients with severe trauma (ISS > 15) and patients with minor injuries (ISS < 15). In these studies, the mean ISS varied between 5 and 12.

**Table 3:** Overview of the correlation between ISS and the occurrence of depression

Author	ISS (Mean ± SD or Median [IQR])	ISS definition for analysis	Depression measurement	Depression measured	Depression definition for analysis	Univariable analysis	Multivariable analysis
Archer et al	11 ± 8	Continuous	PHQ-9	T = 12 m: 56% (PHQ ≥ 10)	PHQ < 10 vs ≥ 10	OR 1.1 (95% CI 0.99—1.2), *P* = > 0.05	n/a
Baecher et al	11 ± 8	1. High severity (centred around one standard deviation above the mean) 2. Moderate severity (centred around the mean) 3. Low severity (centred around one standard deviation below the mean)	HADS	T = 12 m: Female: Mean HADS = 2.0 Male: Mean HADS = 1.8	Continuous	n/a	F(1, 986.17) = 0.55 *P* = 0.46
Boals et al	12 ± 8	Continuous	PHQ-8	T = 24 h: mean PHQ 7.8 T = 1 m: mean PHQ 7.7 T = 6 m: mean PHQ 7.1 T = 12 m: mean PHQ 6.8	Continuous	Pearson correlation: T = 24 h: 0.08, *P* = > 0.05 T = 1 m: 0.03, *P* = > 0.05 T = 6 m: 0.03, *P* = > 0.05 T = 12 m: 0.16, ***P* = < 0.05**	n/a
Bocci et al	29 [22–38]	Continuous	HADS-14	*T* = 12-24 m: 94% (HADS ≥ 11)	Continuous	Spearman correlation: 0.16, P = 0.41	n/a
Biçen et al	18 ± 9	< 16 vs ≥ 16	BDI	*t* = 9 m: 85%	Continuous	P = 0.955	n/a
Gouweloos et al	7 ± 9	≤ 8 vs ≥ 9	PHQ-2	*T* = 2 m: 35% *T* = 9 m: 32% (PHQ ≥ 3)	Continuous	*T* = 2 m: PHQ = 1.8 vs 2.1, *P* = > 0.05 *T* = 9 m: PHQ = 1.6 vs 2.2, *P* = > 0.05	n/a
Halvachizadeh et al	20 ± 9	Continuous	HADS-14	*T* = > 20y 25% Mild, 24% Moderate 24% Severe	HADS < 11 vs ≥ 11	Mean ISS: 21 vs 19 *P* = > 0.05	n/a
Jackson et al	66% ISS > 25	≤ 25 vs > 25	BDI-II	*T* = 12 m: 40%	"Clinically significant depression symptoms"	35% vs 44% depression, *P* = 0.46	n/a
Kumar et al	Mean 7	Continuous	HADS	*T* = 1d: 43% (HADS ≥ 8)	HADS < 8 vs ≥ 8	Mean ISS: 6.4 ± 8.5 vs 7.6 ± 2.6, ***P* = 0.002**	OR 1.13, *P* = 0.095 *
Rainey et al	11 ± 7	Continuous	PHQ-8	*T* = 0: 24% *T* = 12 m: 30% (PHQ ≥ 10:)	Continuous	Pearson correlation: *t* = 0: 0.028, *P* = 0.775 *t* = 12 m: 0.114, *P* = 0.239	n/a
Schweininger et al	11 ± 8	Continuous	HADS	Mean HADS: T = 0: 4.9 T = 3 m: 4.9 T = 12 m: 4.5	Continuous	Regression *T* = 0: 0.02, *P* = > 0.05 *T* = 3: -0.02, *P* = > 0.05 *T* = 12 m: 0.04, *P* = > 0,05	n/a
Shelley et al	9 [5–14]	Continuous	PHQ-8	*T* = 0: 27% *T* = 12 m: 29% (PHQ ≥ 10)	PHQ < 10 vs ≥ 10	*T* = 0: median ISS: 10 vs 9, *P* = 0.136 *T* = 12 m: median ISS: 9 vs 9, *P* = 0.806	n/a
Shih et al	9 ± 7	< 10 vs ≥ 10	PHQ-8	6 m: 31% 12 m: 28% (PHQ ≥ 10)	PHQ < 10 vs ≥ 10	OR 1.53 (95% CI 0.98—2.39) *P* = > 0.05	n/a
Skogstad et al	9 (min 1-max 54)	1–8 vs 9–15 vs 16–24 vs 25–75	HADS	*T* = 12 m: 11% (HADS ≥ 8)	HADS < 8 vs ≥ 8	OR: ISS 1–8: ref ISS 9–15: 2.84 (95% CI 1.02–7.89), ***P* = 0.045** ISS 16–24: 4.23 (95% CI 1.37–13.07)**, *P* = 0.012** ISS 25–75: 1.76 (95% CI 0.3309.51), *P* = 0.510	*P* = > 0.05**
vanDelft-Schreurs et al	21 [17–27]	15–24 vs ≥ 25	HADS	*T* = 15-53 m: 12% (HADS ≥ 11)	HADS < 11 vs ≥ 11	"No significant association between any accident-or injury-related factor and the occurrence of psychological complaints was found"	n/a
Vincent et al	8 ± 5	Continuous	BDI-II	*T* = 12w: 21% (BDI ≥ 20)	BDI < 20 vs ≥ 20	Mean ISS: 7.2 ± 4.5 vs 10.9 ± 6.4, ***P* = 0.016**	n/a
Wu et al	8 ± 5	Continuous	HAMD-17	*T* = 0: 24% (HAMD ≥ 7)	Continuous	Spearman correlation: 0.12, ***P* = 0.016**	*P* = > 0.05***

*IQR* = Interquartile range, *PHQ*: Patient Health Questionnaire, *HADS* = Hospital Anxiety and Depression Scale, *BDI* = Back Depression Inventory, *HAMD* = Hamilton Depression Scale, *ISS* = Injury Severity Score. Significant = Bold

* Significant confounders: younger age, higher pain score, gender (female), being single, nuclear family

** Significant confounders: Low education level, pessimism measured with Life Orientation Test-Revised, dissociation measured by Casualty Chain Inventory

*** Significant confounders: gender (woman), longer length of hospital stay

### Quantification of symptoms of depression

Seven studies used the Hospital Anxiety and Depression Scale (HADS) to measure symptoms of depression (Table 3) [5, 36, 37, 39–42]. The HADS is a 14-item questionnaire, developed in 1983 for measuring symptoms of anxiety and depression [43]. Higher scores indicate greater symptoms of depression.

The Patient Health Questionnaire (PHQ) was used in six studies [44–49]. The original PHQ-9 is a patient-reported questionnaire scoring each of the 9 criteria for depression in the DSM-IV (Diagnostic and Statistical Manual of Mental Disorders) [44]. The PHQ asks patients to rate the frequency and severity of symptoms during the previous two weeks on a scale from 0 (not at all) to 3 (nearly every day), with higher total scores indicating greater symptoms of depression [45, 50]. In the PHQ-8 version, one question regarding suicidal and self-injurious thoughts is removed [51]. Gouweloos et al. used the PHQ-2, which is a shortened, validated, 2-item questionnaire measuring symptoms of depression [46].

Biçen et al. [27] used the Beck Depression Inventory (BDI) and Vincent et al. [52] and Jackon et al. [38] used the Beck Depression Inventory-II (BDI-II) [53, 54], and Wu et al. [54] used the Hamilton Depression Scale-17 (HADS-17) to measure symptoms of depression.

### The association between injury severity and symptoms of depression (Table 3).

#### During hospitalization

Wu et al. [54] found a slight Spearman correlation (Spearman correlation 0.12, *P* = 0.016, *N* = 323) between ISS and presence of symptoms of depression during hospitalization in bivariate analysis that was not retained in a multivariable model, where women and longer length of stay in hospital were associated with associated with greater symptoms of depression. Kumar et al. [5] found a slightly higher ISS among patients with HADS ≥ 8 in bivariate analysis (mean 7.6 ± 2.6 vs 6.4 ± 8.5, *P* = 0.002, *N* = 190) that was not retained in a multivariable analysis where younger age, higher pain score, being single, nuclear family, and women were associated with higher threshold symptoms of depression (Table 3). Four studies found no bivariate association at this time point. [34, 41, 45, 48]

#### Between 2 and 9 months after injury

Of the 6 studies that performed bivariable analyses of the relationship between ISS and symptoms of depressions between 2 weeks and 9 months, only Vincent et al. [52] reported an association; patients with BDI ≥ 20 had higher ISS score at 12 weeks (10.9 ± 6.4 [Standard deviation] vs 7.2 ± 4.5, *P* = 0.016, *N* = 101).

#### Twelve months after injury

Two studies reported an association between ISS and symptoms of depression 12 months after injury and seven did not [19–23, 28, 32, 34, 35]. Boals et al. [45] noted a slight correlation (Pearson correlation coefficient 0.16, *p* =  < 0.05) and did not perform multivariable analysis. Skogstad et al. [23] used logistic regression for bivariate analysis and found an association between ISS and symptoms of depression with modest odds ratios, but wide confidence intervals (ISS 1–8: ref; ISS 9–15: Odds Ratio (OR) 2.8 (95% CI 1.0 – 7.9), *p* = 0.045; ISS 16–24: OR 4.2 (95% CI 1.4 – 12.1), *p* = 0.012) that was not retained in multivariable analysis.

#### More than 12 months after injury

Three studies analyzed the association between ISS and depressive symptoms more than 12 months after injury and found no association. Bocci et al. [36] contacted 32 patients between 12 and 24 months after ICU discharge and found no correlation between ISS and HADS. In their study group of severely injured patients (ISS ≥ 16), Halvachizadeh et al. [37] found no statistically significant difference in ISS among patients with HADS < 11 vs ≥ 11 (ISS 21 vs 19) after more than 20 years. Van Delft-Schreurs et al. [39] sent questionnaires by post, which were completed between 15 and 53 months after trauma and also did not find an association (depression measured with HADS).

## Discussion

Although there is an increasing body of evidence that symptoms of depression are common after physical trauma, and that psychological factors have a notable association with physical outcomes, little is known about association of the severity of physical trauma with symptoms of depression during recovery. In this systematic review of studies of the association of symptoms of depression and ISS there was little or no correlation.

There are limitations to consider. Many of the studies included in this systematic review had methodological shortcomings. For example, none of the studies reported a loss to follow-up less than 5%, which may have caused a non-response bias. All studies except one were performed in western countries. The methods of all the studies were disparate, and we could not perform a meta-analysis. Half of the studies dichotomized symptoms of depression for the bivariate analysis with the ISS and they used various instruments and variable threshold scores. In our opinion, dichotomizing a continuous mental health measure loses information, increases the potential for introducing bias, and could reinforce a false mind–body dichotomy and social stigma associated with mental health. Another reason to analyze symptoms of depression on a continuum is that major depression can only be diagnosed by a psychiatrist after evaluation, and estimates based on a threshold may be misleading in this regard. However, despite the heterogeneity in study designs and outcome measures, the studies showed no or only small correlations, where one might intuitively expect a large correlation. More research is needed which should preferably be prospective studies including large cohorts of trauma patients, with symptoms of depression on a continuum Also, it will be possible to obtain different subjective scores for trauma, for example on the severity and the mechanism, and other psychological outcomes.

The observation that symptoms of depression are not associated with injury severity in studies using various measures in various settings suggests that clinicians should anticipate symptoms of depression in all people recovering from physical trauma, not just people with relatively severe injury. It could be that the circumstances of a trauma are more relevant than the severity in the experience of symptoms of depression. For instance, Gouweloos et al. [46] included patients after a commercial airplane crash. After 2 and 9 months, they found, respectively, 32 and 35% of the patients were at risk for a depression, measured with the PHQ-2 [46]. Biçen et al. [27] included patients after injuries from the Libyan Civil War. At mean time of 9 months after injury, they found a high prevalence: 85% had symptoms of depression defined as a score greater than 14 on the BDI.

It could also be that other characteristics of an injury are associated with symptoms of depression. Gouweloos et al. [46] and Ahl et al. [3] found a correlation between length of hospital stay and symptoms of depression, but Biçen et al. [27] did not find this correlation. Ahl et al. [55] found penetrating trauma was associated with post-traumatic prescribed antidepressant within a year. Furthermore, it is possible that ISS and other characteristics of the injury have limited associations with levels of depression compared to personal factors. The majority of included patients were men (weighted mean 70%). A meta-analysis with 58 included studies found masculinity as a protective factor for depression, less so at older ages [56]. Also in this systematic review, Wu et al. [54] and Kumar et al. [5] found women were associated with greater symptoms of depression in multivariable analysis.

## Conclusion

From the results of this systematic review, we conclude that there is little or no association between severity of physical trauma reflected by the ISS, and symptoms of depression. The relationship between depressive symptoms and other patient and injury characteristics is, at best, weak and inconsistent, so action should be taken to further explore the relationship between symptoms of depression after trauma and psychological or injury-specific characteristics, as well as to propose early measures to prevent depression among trauma patients. For now, clinicians can anticipate symptoms of depression among all trauma patients given the evidence that both minor and major trauma have a notable risk of developing symptoms of depression, which may also lead to worse physical outcomes [3, 7, 9–11, 57].

## Appendix 1

### Search strategy PubMed

("Wounds and Injuries"[Mesh] OR Trauma*[tw] OR Injur*[tw] OR "Multiple Trauma"[Mesh] OR Polytrauma[tw]) AND ("Depression"[Mesh] OR Depression[tw] OR Depressive Symptom*[tw] OR "Depressive Disorder"[Mesh] OR Depressive Disorder*[TW]) AND ("Injury Severity Score"[Mesh] OR "Injury Severity Score"[TW] OR ISS Score*[TW] OR Injury score*[tw] OR "injury severity” [TW]) NOT neuro* [ti] NOT brain* [ti].

### Search strategy Embase

(exp *injury/ OR Trauma*.ti. OR Injur*.ti. OR Polytrauma.ti.) NOT "brain".ti. NOT "neuro".ti. NOT "head".ti. NOT "spinal cord".ti. NOT "TBI".ti. AND (exp *depression/ OR Depression.mp. OR Depressive.mp.) AND ( exp injury scale/ OR "Injury Severity Score".mp. OR "ISS".mp. OR "injury severity".ab. OR "injury characteristics".ab.) NOT (conference OR conference abstract OR "conference review").pt. NOT "child*".ti. NOT "adult*".ti. AND 2010:2021.(sa_year).
